# Self-care practice and associated factors among patients with diabetes on follow-up at Yirgalem General Hospital, Sidama, Ethiopia: a cross-sectional study

**DOI:** 10.1186/s12902-024-01647-9

**Published:** 2024-07-11

**Authors:** Mehreteab Million Kobamo, Fanuel Belayneh Bekele, Yilkal Simachew, Mahlet Tesfaye Abebe, Kibruyisfaw Weldeab Abore

**Affiliations:** 1 KNCV Tuberculosis Foundation, Hawassa, Ethiopia; 2 School of Public health, College of Medicine and Health Science, Hawassa University, Hawassa, Ethiopia; 3 Yirgalem General Hospital, Yirgalem, Sidama Ethiopia; 4 Yirgalem Hospital Medical College, Yirgalem, Sidama Ethiopia

**Keywords:** Diabetes, Diabetes Mellitus, Self-care, Self-management, Ethiopia

## Abstract

**Background:**

Self-care practice is an integral and efficient part of comprehensive diabetes management, which could be influenced by various socio-demographic, clinical, and lifestyle factors.

**Objective:**

The study aimed to assess the level of diabetes self-care practice and its associated factors among patients with diabetes on follow-up at Yirgalem General Hospital, Yirgalem, Sidama, Ethiopia.

**Methodology:**

An Institution-based cross-sectional study was conducted from February 15 to May 10, 2022, involving 298 patients with diabetes on follow-up at Yirgalem General Hospital. A pre-tested interviewer-administered questionnaire was utilized to collect data from patients. A descriptive analysis was conducted to determine the level of good self-care practice. Bivariate and multivariable binary logistics regression were performed to determine factors associated with good diabetic self-care practice. Associations with a *p*-value < 0.05 were considered statistically significant.

**Result:**

The overall good diabetic self-care practice among patients was 59.4%. Regarding the specific domains of care, 15 (5%) participants had good self-glucose monitoring care, 228 (76.5%) had good exercise self-care, 268 (89.9%) had good dietary self-care, 228 (76.5%) had good foot self-care, and 260 (87.2%) had good diabetic medication adherence. Single marital status (AOR = 5.7, 95% CI: (1.418, 22.915), urban residence (AOR = 2.992, 95% CI: (1.251, 7.153)), and having a glucometer (AOR = 2.273, 95% CI: (1.083, 4.772)) were factors that were significantly associated with good diabetic self-care practice.

**Conclusion:**

Good diabetic self-care practices among participants was low. Marital status, place of residence, and having a glucometer were statistically significant predictors of good diabetic self-care practices. Targeted intervention addressing those patients from rural areas to increase awareness and practice of self-care, as well as the promotion of having a glucometer at home for self-glucose monitoring is recommended.

**Supplementary Information:**

The online version contains supplementary material available at 10.1186/s12902-024-01647-9.

## Introduction

Diabetes mellitus (DM) is a prevalent chronic metabolic disorder that is manifested by hyperglycemia. If not properly controlled, it would cause damage to the heart, blood vessels, eyes, kidneys, and nerves [1]. Globally, more than half a billion people are estimated to be affected by diabetes. Low-income countries are among the highly affected regions of the world, with a concomitant high burden of undiagnosed DM [2]. The World Health Organization (WHO) estimates the prevalence of diabetes in Ethiopia to be 3.8%, which is among the highest in Africa [3]. In Ethiopia, most people with diabetes remain undiagnosed until serious complications become evident.

Diabetes poses a great risk to the health and well-being of the population and the economy [4]. To minimize the risk of diabetic complications and the subsequent cost of treatment, patients need to achieve good glycemic control. However, regardless of various scientific breakthroughs and newer innovations in the pharmaceutical arena, good diabetic control remains a paramount challenge to halting this metabolic pandemic [5]. This incoherence reflects the central role that individuals play in determining their diabetes control status. Self-care practices in diabetes is central and irreplaceable to keep the illness under control, and much of the care is provided by the patients themselves [6, 7]. Self-managing the disease includes following a prescribed medication regimen, a strict calorie-controlled diet, doing regular exercise, undertaking blood glucose checks, and caring for feet [8–10]. Ongoing diabetes self-management education and support are critical to preventing acute complications and reducing the risk of long-term complications [11]. Empowering patients to adhere to good diabetic self-care practices is critical in the management of patients with diabetes. Evidence from previous studies also showed that self-management training in Type 2 diabetes is effective for short-term glycemic control [12, 13].

Previous studies conducted in Ethiopia showed that the overall prevalence of good diabetes self-care behavior among patients with diabetes ranged from 28.4 to 76.8% [14–16]. This wide variation in self-care practice is expected from a country like Ethiopia, which has socioeconomic and cultural varieties. Therefore, generating local evidence that corresponds to those contextual differences is crucial. Moreover, most studies conducted in Ethiopia assessed the level of diabetes self-care practices among patients aged 18 years or older and did not include adolescents. This resulted in gaps in considering patients with type 1 diabetes. In addition, only a limited number of studies have assessed the role of diabetes self-care education and family support. Therefore, this study aims to assess the level of diabetic self-care practice and its associated factors among patients with DM on follow-up at Yirgalem General Hospital.

## Method

### Study setting and study design

The study was conducted at Yirgalem General Hospital (YGH). The hospital is located in Yirgalem town, Sidama National Regional State, Ethiopia, 325 km from the national capital, Addis Ababa city. The hospital serves an estimated 1.5 million people in its catchment area. The diabetic clinic has 914 registered patients with diabetes on follow-up. An Institution-based cross-sectional study was conducted at YGH from February 15 to May 10, 2022.

### Study population

The study population for this study were all patients with diabetes aged ≥ 15 years old and on follow-up at the diabetic clinic of YGH who visited during the data collection period.

### Inclusion and exclusion criteria

#### Inclusion criteria

All patients with diabetes aged ≥ 15 years old who were on follow-up for at least six months at YGH were eligible for this study.

#### Exclusion criteria

Patients with diabetes who were critically ill and unable to communicate and those patients who revisited during the data collection period once they had been included in the study were excluded from the study.

### Sample size and sampling procedure

The sample size was determined using Epi Info version 7 software using the following assumptions: 95% confidence interval (95% CI), 5% margin of error (d), 53.3% level of good diabetic self-care [17], and *N* = 914. After accounting for a 10% non-response rate, a sample size of 298 was obtained.

A systematic random sampling technique was employed to select study subjects using a sampling interval of K = 3. When a patient didn’t fulfill the inclusion criteria, the next patient who fulfilled the criteria was included in the study. Patient’s medical record numbers were listed accordingly and signed to avoid patient reselection if repeated visits occurred during the study period. Participants were interviewed after they completed refill of their medications.

### Data collection procedure and quality control

Data were collected using an interviewer-administered questionnaire that assessed socio-demographic data, clinical characteristics, and lifestyle information. The level of diabetes self-care practice was assessed using the Summary of Diabetes Self-Care Activities (SDSCA), which includes a healthy diet, physical exercise, blood sugar testing, foot care, and smoking [18]. All questions were prepared in English, translated into Amharic, and then back translated to English to check their consistency. A pre-test of the data collection tool was conducted on 5% of the sample size at Adare General Hospital to identify potential gaps.

Data were collected by two trained nurses and supervision was done by a nurse with a bachelor’s degree. Two days of training and practical demonstrations on interview techniques and measurement procedures were given to data collectors. Data collection was done after informed consent was obtained. After interviews were completed, data from patient charts regarding comorbidities and diabetic complications was collected. Regular supervision and check-up of the completeness, consistency, and clarity of responses to questions were done by the investigators and the supervisor.

### Data processing and analysis

Data entry, coding, and verification were conducted using Epidata software version 3.1. The data were exported to SPSS v.23 for analysis. Descriptive summary using proportion, frequency distribution, and mean with a standard deviation was done. Logistic regression was computed to identify factors associated with good diabetes self-care practice. Those variables with a *p*-value of < 0.25 on bivariate logistic regression were included in the multivariable logistic regression model to adjust for possible confounding effects. The model’s goodness of fit was assessed using the Hosmer and Lemshow goodness of fit test. Associations were measured using an adjusted odds ratio (AOR) with a 95% confidence interval (CI), and those with a *p*-value < 0.05 were considered statistically significant.

### Operational definitions

Diabetes self-care practice: Five domains of self-care practices (diet, exercise, foot care, blood glucose testing, and smoking) were used to assess the self-care practices. For all domains, the frequency of self-care activity in the last 7 days was measured. For each domain, the score was calculated and categorized as good or poor [18, 19].

Drug adherence: patients were labeled as having good adherence if they took at least 80% of the prescribed medication for the week [20].

Dietary self-care: patients were classified as having good dietary self-care practice if they scored above or equal to the mean score [21, 22].

Exercise self-care: patients were labeled as having good exercise self-care practice if they scored above or equal to the mean score on exercise self-care questions [21, 22].

Glucose monitoring: patients were labeled as having good self-glucose monitoring practice if they scored above or equal to the mean score on glucose monitoring self-care questions [21, 22].

Foot care: patients were labeled as having good foot care practice if they scored above or equal to the mean score on foot self-care questions [21, 22].

The overall score was calculated by summing the mean score for diet, exercise, foot care, and blood glucose testing divided by five. After calculating an overall mean score, patients were classified as having good self-care practices if they scored above or equal to the mean score [18, 21].

Diabetic Knowledge: it was assessed as a composite variable of 16 questions regarding various aspects of diabetes and diabetes self-care. Correct responses were scored ‘1’ and incorrect responses were scored ‘0’. After summing up each score, patients were classified as having good knowledge if they scored above or equal to the mean knowledge score [16, 23].

## Results

### Socio-demographic characteristics

In this study, nearly half of the participants (41.9%) were within the age category of 25–40, 165 (55.4%) were male, and 133 (44.6%) were married. Furthermore, 85 (28.5%) participants had a college education and 88(29.5%) were civil servants. More than half of the respondents (155: 52%) had a monthly income of less than 1500 Ethiopian birr (ETB), while only 17 (5.7%) had a monthly income of 7500 ETB or more. The majority of respondents were protestant in religion (170: 57%) and rural dwellers (156: 52.3%) (Table 1).

**Table 1:** Socio-demographic characteristics of patients with diabetes on follow-up at YGH, 2022

Variables		Frequency (*N* = 298)	Percentage (%)
Age group			
	< 25	65	21.8
	25–40	125	41.9
	41–55	51	17.2
	> 55	57	19.1
Sex			
	Male	165	55.4
	Female	133	44.6
Marital status			
	Single	108	36.2
	Married	133	44.6
	Divorced	27	9.1
	Widowed	30	10.1
Education status			
	Non-literate	81	27.2
	Primary school	66	22.1
	High school	66	22.1
	College and above	85	28.5
Occupation			
	Farmer	82	27.5
	Civil servant	88	29.5
	Merchant	40	13.4
	Daily laborer	34	11.4
	Housewife	39	13.1
	Pension	15	5
Income			
	< 1500	155	52
	1500–3499	43	14.4
	3500–5499	61	20.5
	5500–7499	22	7.4
	≥ 7500	17	5.7
Religion			
	Orthodox	83	27.9
	Protestant	170	57
	Muslim	45	15.1
Residence			
	Urban	142	47.7
	Rural	156	52.3

### Clinical and behavioral characteristics of respondents

In this study, 152 (51%) of respondents had Type-2 diabetes and more than two-thirds of the subjects (214: 71.8%) had no family history of diabetes. Moreover, 191 (64.1%) patients were on insulin, while 67 (22.5%) were on oral medication. The majority of patients (199: 66.8%) responded that they had been on treatment for the past 3–6 years. Meanwhile, the mean fasting blood sugar for the participants on the day of the interview was 173 mg/dl (SD = ± 70). Regarding follow-up, a higher proportion of patients (237: 79.5%) responded that they had follow-up every month. Furthermore, around a third of the participants (101: 33.9%) had diabetic complications. Diabetic neuropathy (51: 50.2%) was the most common complication among respondents who had diabetic complications. In addition, 80 (26.8%) had associated comorbidity, and more than one-third (145: 33.2%) of the respondents had prior hospital admissions. Around half of the participants (115: 48.7%) responded that they have a functioning glucometer at home. Meanwhile, 42 (14.1%) of the study participants reported that they consume alcohol, and 9 (3%) reported they smoked at least one cigarette in the past 7 days (Table 2).

**Table 2:** Clinical and behavioral characteristics of patients with diabetes on follow-up at YGH, Yirgalem, 2022

Factors		Frequency	Percentage
Diabetes type			
	Type 1	146	49
	Type 2	152	51
Family history			
	Yes	84	28.2
	No	214	71.8
Treatment type			
	Oral	67	22.5
	Insulin	191	64.1
	Mixed	40	13.4
Duration of treatment			
	< 3years	37	12.4
	3–6 years	199	66.8
	7–10 years	53	17.8
	> 10 years	9	3
Today’s FBS (Mean ± SD)		173	70
Frequency of follow-up			
	Every 2 weeks	58	19.5
	Monthly	237	79.5
	Every 2 month	3	1
Presence of diabetic complication			
	Yes	101	33.9
	No	197	66.1
Complication type			
	Retinopathy	14	13.9
	Nephropathy	13	12.9
	Neuropathy	51	50.5
	Coronary artery disease	9	8.9
	Peripheral arterial disease	14	13.9
Presence of comorbidity			
	Yes	218	73.2
	No	80	26.8
Prior admission			
	Yes	145	33.2
	No	153	66.8
Glucometer Ownership			
	Yes	115	38.6
	No	183	61.4
Alcohol consumption			
	Yes	42	14.1
	No	256	85.9
Smoking			
	Yes	289	97
	No	9	3

### Psychosocial characteristics of respondents

Of the respondents, 287 (96.3%) reported that they had received diabetic self-management education (DSME). In addition, among those who received education, 176 (61.3%) of them responded that they believed the education was helpful. Meanwhile, 276 (92.6%) of the subjects reported that they had family support. Illness-related support (224: 81.2%) was the most common followed by psychological support (42: 15.2%). Furthermore, more than half (160; 58%) of the participants reported that they had strong family support (Table 3).

**Table 3:** Clinical and psychosocial support profile of patients with diabetes at YGH, 2022

Variables		Frequency	Percentage
DSME received			
	Yes	287	96.3
	No	11	3.7
Was DSME helpful			
	Strongly agree	176	61.3
	Slightly	110	38.3
	Strongly disagree	1	0.4
Family support			
	Yes	276	92.6
	No	22	7.4
Family Support type			
	Psychological	42	15.2
	Illness related	224	81.2
	Financial	10	3.6
Rating of family support			
	Very strong	55	19.9
	strong	160	58
	Moderate	49	17.8
	Minimal	9	3.3
	poor	3	1.1

DSME: diabetes self-management education

### Knowledge of diabetes

In this study, only 161 (54%) had good knowledge of diabetes.

### Self-care practice among patients with diabetes

In this study, 15 (5%) had good self-glucose monitoring care, 228 (76.5%) had good exercise self-care, 268 (89.9%) had good dietary self-care, 228 (76.5%) had good foot self-care, and 260 (87.2%) had good diabetic medication adherence (Table 4). The overall self-care practice was found to be good in 177 (59.4%) of the respondents.

**Table 4:** Self-care practices across the five domains among patients with diabetes in YGH, 2022

Variables	Good care	Poor care
Self-glucose monitoring	15(5%)	283(95%)
Exercise	228(76.5%)	70(23.5%)
Dietary self-care	268(89.9%)	30(10.1%)
Foot care	228(76.5%)	70(23.5%)
Drug adherence	260(87.2%)	38(12.8%)

After performing bivariate logistic regression, age, marital status, education level, occupation, income category, religion, residence area, duration of treatment, frequency of follow-up, presence of diabetic complications, comorbidity status, having a glucometer, and diabetic knowledge were included in the multivariable binary logistic regression model (Table 5).

**Table 5:** Bivariate and multivariable analysis of factors associated with self-care practice among patients with diabetes on follow-up at YGH, 2022

Variables		Good	Poor	COR (95%C.I)	AOR (95%CI)	*p*-value
Age group						0.526
	< 25	47(72.3%)	18(27.7%)	3.59 (1.686,7.647)	1.933(0.384,9.734)	0.424
	25–40	82(55.6%)	43(34.4%)	2.622(1.379,4.985)	1.267(0.372,4.315)	0.705
	41–55	24(47.1%)	27(52.9%)	1.222(0.571,2.616)	1.97(0.695,5.581)	0.202
	> 55	24(42.1%)	33(57.9%)	1.00	1.00	
Marital status						**0.009** ^*^
	Single	83(76.9%)	25(23.1%)	5.735(2.41,13.64)	5.7(1.418,22.915)	0.014
	Married	60(45.1%)	73(54.9%)	1.42(0.627,3.215)	0.996(0.36,2.76)	0.994
	Divorced	16(85.2%)	11(14.8%)	9.932(2.718,36.286)	3.251(0.61,17.343)	0.168
	Widowed	11(36.7%)	19(63.3%)	1	1	
Education level						0.619
	Non-literate	30(37%)	51(63%)	0.136 (0.067,0.276)	1.969(0.339,11.43)	0.45
	Primary school	30(45.5%)	36(54.5%)	0.193 (0.093,0.4)	0.995(0.201,4.912)	0.995
	High school	48(72.7%)	18(27.3%)	0.618(0.287,1.332)	1.114(0.265,4.686)	1.114
	College	69(81.2%)	16(18.8%)	1.00		
Occupation						0.153
	Farmer	37(45.1%)	45(54.9%)	0.548(0.179,1.681)	4.36(0.51,37)	0.177
	Civil servant	74(84.1%)	14(15.9%)	3.524(1.08,11.47)	5.42(0.889,33.04)	0.067
	Merchant	27(67.5%)	13(32.5%)	1.385(0.4,4.722)	3.33(0.392,28.31)	0.27
	Daily laborer	21(61.8%)	13(38.2%)	1.077(0.311,3.733)	1.99(0.223,17.94)	0.536
	Housewife	9(23.1%)	30(76.9%)	0.2 (0.056,0.715)	1.947(0.168,22.57)	0.594
	Pension	9(60%)	6(40%)	1.00		
Income level						0.789
	< 1500	68(43.9%)	87(56.1%)	0.49 (0.006,0378)	0.219(0.016,3.037)	0.258
	1500–3499	29(67.4%)	14(32.6%)	0.129(0.016,1.077)	0.307(0.024,3.933)	0.364
	3500–5499	49(80.3%)	12(19.7%)	0.255(0.031,2.119)	0.397(0.035,4.559)	0.458
	5500–7499	15(65.2%)	7(34.8%)	0.134(0.015,1.221)	0.282(0.02, 3.588)	0.329
	≥ 7500	16(94.1%)	1(5.9%)	1.00		
Religion						0.427
	Orthodox	65(78.3%)	18(21.7%)	2.639(1.199,5.80)	1.241(0.373,4.126)	0.725
	Protestant	86(50.6%)	84(49.4%)	0.748 (0.385,1.453)	0.694(0.295,1.629)	0.401
	Muslim	26(57.8%)	19(42.2%)	1.00		
Residence						**0.014** ^*^
	Urban	112(78.9%)	30(21.1%)	5.227(3.128,8.734)	2.992(1.251,7.153)	
	Rural	65(41.7%)	91(58.3%)	1		
Duration of treatment						0.18
	< 3years	25(67.6%)	12(32.4%)	0.26(0.029,2.327)	0.099(0.007,1.373)	0.085
	3–6 years	124(62.3%)	75(37.7%)	0.207(0.025,1.685)	0.08(0.007,0.967)	0.047
	7–10 years	20(37.7%)	33(62.3%)	0.076(0.009,0.652)	0.061(0.005,0.764)	0.03
	> 10 years	8(88.9%)	1(11.1%)	1		
Follow-up frequency						
	Every 2 week	50(86.2%)	8(13.8%)	5.561(2.528,12.23)	1.706(0.573,5.074)	0.337
	Monthly	127(52.9%)	113(47.1%)	1.00		
DM complication						
	Yes	50(50.5%)	51(49.5%)	0.54 (0.322,0.88)	0.888(0.386,2.041)	0.78
	No	127(64.5%)	70(35.5%)	1.00		
Comorbidity						
	Yes	40(50%)	40(50%)	1.69 (1.008,2.837)	0.669(0.259, 1.727)	0.406
	No	137(62.8%)	81(37.2%)	1.00		
Own glucometer						
	Yes	91(79.1%)	24(20.9%)	4.227(2.504,7.304)	2.273(1.083,4.772)	**0.03** ^*^
	No	86(47%)	97(53%)	1.00		
Diabetic knowledge						
	Good	110(68.3%)	51(31.7%)	2.253(1.406, 3.611)	1.996(0.994,4.009)	0.052
	Poor	67(48.9%)	70(51.1%)	1.00		

^*^P < 0.05

After adjusting for other variables, marital status, residence area, and having a glucometer at home were found to be statistically significant predictors of self-care practice among patients with diabetes. The model’s goodness of fit was assessed using the Hosmer and Lemshow goodness of fit test and it fitted (*p*-value = 0.285).

It was found that patients with diabetes who were single had 5.7 times higher odds of having good diabetic self-care practices than those who were widowed (AOR = 5.7, 95% CI: 1.418, 22.915). Among patients with diabetes, those who reside in urban areas had 2.99 times higher odds of having good diabetic self-care practice than those living in rural areas (AOR = 2.992, 95% CI: 1.251, 7.153). It was also noted that those who have a glucometer at home had 2.27 times higher odds of having good diabetic self-care practices than those who don’t (AOR = 2.273, 95% CI: 1.083, 4.772) (Table 5).

## Discussion

In this study, the overall good diabetic self-care practice was 59.4%. This finding is comparable with a study done in West Ethiopia (60.7%) [22], but lower than a study done in southern Ethiopia (76.8%) [16] and in Iran (73.8%) [24]. However, it is higher than reported by a study done in Northwest Ethiopia (28.4%) [25]. This could be due to variations in the socio-demographic and cultural characteristics of the study populations.

In addition, marital status, place of residence, and having a glucometer were found to be statistically significant predictors of good self-care practice after adjusting for other variables. The study showed that those patients who are single were more likely to have good practice than those who are widowed (AOR = 5.7, 95% CI: 1.418, 22.915). This result was not reported in previous studies done in various parts of Ethiopia. Furthermore, patients with diabetes who are from urban areas were found to have higher odds of good self-care practice than those from rural areas (AOR = 2.992, 95% CI: 1.251, 7.153). This finding is supported by a study done at Nekemte referral hospital in western Ethiopia, which showed that those patients who live in urban areas had 5.5 times higher odds of good self-care practice (AOR = 5.517, 95% CI:2.184–13.938) [22]. Moreover, a multicenter study done in the Tigray region of Ethiopia also showed that those patients who are from urban areas had 1.9 times higher odds of good self-care practice (AOR = 1.9, 95% CI: 1.20–2.94) [21]. This finding could be explained by the fact that those patients living in urban areas have higher access to health facilities, including pharmacies and clinics, which would enable easy monitoring of glycemic levels [21, 22].

This study also showed that patients with diabetes who have a glucometer at home were more likely to have good diabetic self-care practices than those who don’t (AOR = 2.273, 95% CI: 1.083, 4.772). This finding is supported by a study done in West Ethiopia, which reported that those who don’t have access to self-glucose monitoring had 9.448 times higher odds of poor diabetic self-care practice (AOR = 9.448, 95% CI: 2.198–40.617) [22]. Furthermore, a study done in a diabetic clinic at Ayder referral hospital showed that patients with diabetes who have access to self-glucose monitoring had 3.7 times higher odds of good diabetic self-care practice than those who do not (AOR = 3. 719, 95%CI: 1.7, 8.139) [26]. This finding could be related to the high likelihood of frequent glycemic level monitoring and glycemic awareness among patients who have a glucometer at home which would promote lifestyle changes in favor of good self-care practice [21, 22, 26].

### Limitations of the study

This study was not without limitations. First, data were collected using an administered questionnaire, which could have introduced interviewer bias as well as social desirability bias. This might have led to an overestimation of desirable qualities such as dietary care, exercise, and drug adherence while underestimating the level of non-desirable qualities such as alcohol consumption and cigarette smoking. This could have also resulted in the higher level of knowledge observed in this study. Furthermore, self-care practices were assessed retrospectively and this could have introduced recall bias. Secondly, the study was conducted in a single hospital, which limits the generalizability of the results outside the study area.

## Conclusion

The overall good diabetic self-care practice in this study was low. Marital status, place of residence, and ownership of a functional glucometer were found to be statistically significant predictors of good diabetic self-care practice. Strengthening targeted diabetic education sessions to address gaps in the knowledge and practice of diabetic self-care among rural patients is recommended. In addition, diabetic education should be provided in a local language that they can easily comprehend.

Moreover, providing affordable glucometers with easily accessible glucometer strips in partnership with stakeholders should be done. This would significantly improve self-glucose monitoring practices among patients with diabetes. Furthermore, teaching those who own a glucometer at home to increase their knowledge on the proper utilization of a glucometer is also recommended.

### Electronic supplementary material

Below is the link to the electronic supplementary material.

Supplementary Material 1

## Data Availability

The datasets used for this study are included in the supplementary information files.

## Abbreviations

AOR: Adjusted Odds Ration
DM: Diabetes mellitus
DSCP: Diabetes self-care practice
DSM: Diabetes self-management
DSME: Diabetes self-management education
FBS: Fasting blood sugar
SCP: Self-care practice
SDSCA: Summary of diabetes self-care activity tool
SPSS: Statistical package for social sciences
WHO: World Health Organization
YGH: Yirgalem general hospital
